# Establishment and Validation of Prognostic Nomograms Based on Serum Copper Level for Patients With Early-Stage Triple-Negative Breast Cancer

**DOI:** 10.3389/fcell.2021.770115

**Published:** 2021-11-25

**Authors:** Fangfang Duan, Jianpei Li, Jiajia Huang, Xin Hua, Chenge Song, Li Wang, Xiwen Bi, Wen Xia, Zhongyu Yuan

**Affiliations:** ^1^ Departments of Medical Oncology, The State Key Laboratory of Oncology in South China, Collaborative Innovation Center for Cancer Medicine, Sun Yat-sen University Cancer Center, Guangzhou, China; ^2^ Departments of Clinical Laboratory Medicine, The State Key Laboratory of Oncology in South China, Collaborative Innovation Center for Cancer Medicine, Sun Yat-sen University Cancer Center, Guangzhou, China

**Keywords:** serum copper level, early-stage triple-negative breast cancer, prognostic nomograms, maximally selected log-rank statistics, survival

## Abstract

**Background:** Altered copper levels have been observed in several cancers, but studies on the relationship between serum copper and early-stage triple-negative breast cancer (TNBC) remain scare. We sought to establish a predictive model incorporating serum copper levels for individualized survival predictions.

**Methods:** We retrospectively analyzed clinicopathological information and baseline peripheric blood samples of patients diagnosed with early-stage TNBC between September 2005 and October 2016 at Sun Yat-sen University Cancer Center. The optimal cut-off point of serum copper level was determined using maximally selected log-rank statistics. Kaplan-Meier curves were used to estimate survival probabilities. Independent prognostic indicators associated with survival were identified using multivariate Cox regression analysis, and subsequently, prognostic nomograms were established to predict individualized disease-free survival (DFS) and overall survival (OS). The nomograms were validated in a separate cohort of 86 patients from the original randomized clinical trial SYSUCC-001 (SYSUCC-001 cohort).

**Results:** 350 patients were eligible in this study, including 264 in the training cohort and 86 in the SYSUCC-001 cohort. An optimal cut-off value of 21.3 μmol/L of serum copper was determined to maximally divide patients into low- and high-copper groups. After a median follow-up of 87.1 months, patients with high copper levels had significantly worse DFS (*p* = 0.002) and OS (*p* < 0.001) than those with low copper levels in the training cohort. Multivariate Cox regression analysis revealed that serum copper level was an independent factor for DFS and OS. Further, prognostic models based on serum copper were established for individualized predictions. These models showed excellent discrimination [C-index for DFS: 0.689, 95% confidence interval (CI): 0.621–0.757; C-index for OS: 0.728, 95% CI: 0.654–0.802] and predictive calibration, and were validated in the SYSUCC-001 cohort.

**Conclusion:** Serum copper level is a potential predictive biomarker for patients with early-stage TNBC. Predictive nomograms based on serum copper might be served as a practical tool for individualized prognostication.

## Introduction

Triple-negative breast cancer (TNBC), characterized by the absence of estrogen receptor (ER), progesterone receptor (PR), and human epidermal growth factor receptor 2 (HER-2), is a heterogenic and aggressive subtype of breast cancer. TNBC is associated with a high risk of early relapse, a high degree of invasiveness, and poor prognosis (Kim et al., 2018; Wang et al., 2021). Early-stage TNBC, defined as staged at I-III according to the American Joint Committee on Cancer (AJCC) 2016 (eighth edition) staging criteria (Amin et al., 2017), accounts for 15–20% of all newly diagnosed cases of early breast cancer (Dawson et al., 2009), and its treatment mainly depends on chemotherapy. Despite recent therapeutic advances, nearly 30% of patients with early-stage TNBC remain to develop disease progression within 3–5 years after the diagnosis despite receiving standard chemotherapy (Haffty et al., 2006; Dent et al., 2007; Chaudhary, 2020). Therefore, the identification of effective prognostic markers and development of individualized therapeutic strategies for TNBC patients are critical and necessary.

Copper is an essential element involving in many biological functions, including protein formation, enzyme activation, oxidation-reduction reactions, immunity development, and signaling pathways (Denoyer et al., 2015; Setty et al., 2008; Bhattacharjee et al., 2017). Copper deficiency has been associated with multiple diseases, such as Menkes disease, mild occipital horn syndrome, and bone marrow suppression (Brewer et al., 2000; Tang et al., 2006; Kaler, 2011; Chan et al., 2017). In addition, excessive copper is also harmful and related to diseases like Wilson disease, lymphoma, breast, colon, and lung cancers (Terada et al., 1999; Gupte and Mumper, 2009; Juloski et al., 2020). Copper chelators have been explored to decrease cell proliferation, angiogenesis, tumor growth, and reverse epithelial-mesenchymal transition (Ishida et al., 2013; Brady et al., 2014; MacDonald et al., 2014; Chan et al., 2017). Furthermore, increased serum copper levels have been associated with disease progression or drug resistance in several malignancies, including advanced breast cancer (Mookerjee et al., 2006; Gupte and Mumper, 2009; Majumder et al., 2009; Kaiafa et al., 2012). These findings suggest that the serum copper level might be served as a biomarker for monitoring tumor progression and treatment efficacy. However, the clinical value of serum copper in patients with early-stage TNBC is poorly investigated.

To address this need, we aimed to explore the relationship between baseline serum copper levels and the survival prognosis of patients with early-stage TNBC in this study, furtherly, we hoped to develop prognostic models combining serum copper levels and clinicopathological factor for individualized survival predictions and personalized decision-making.

## Methods and Patients

### Study Design and Patient Eligibility

This study retrospectively analyzed the relationship between serum copper levels and the prognosis of patients with newly diagnosed TNBC at Sun Yat-sen University Cancer Center (SYSUCC) between September 2005 and October 2016. The study was approved by the ethics committee of SYSUCC (Registration number: B2021-218-01), written informed consent from patients was waived because of the retrospective nature of current study and the anonymous processing of patient information. We covered all personal data confidentially and conducted this study in accordance with the Declaration of Helsinki.

Patients were eligible for inclusion in this study if they met the following criteria: 1) women with pathologically diagnosed breast cancer without distant metastasis (e.g. brain, bone, live, lung) at the time of diagnosis, 2) age ≥18 years old, 3) ER/PR-negative, which was considered as <1% positive cells by immunohistochemistry (IHC) staining; HER2-negative tumor, which was defined as a score of 0 or 1 + on IHC or a score of 2 + on IHC without ERBB2 gene amplification on fluorescence *in situ* hybridization, 4) early-stage tumor, i.e., T1-4N0-3M0 according to the AJCC 2016 criteria (eighth edition) (Amin et al., 2017), and 5) availability of complete clinicopathological data and peripheric blood samples obtained within 1 week of the diagnosis for serum copper detection. Key exclusion criteria included: 1) pregnancy or lactation, 2) a history of breast cancer and other primary tumors, 3) severe or uncontrolled infection, and 4) severe metabolic disorder.

### Sample Collection and Serum Copper Measurement

Patients’ clinicopathological data were hand-retrieved from the electronic medical records system of SYSUCC. The peripheric blood samples collected at the time of diagnosis and before the initiation of any anti-cancer treatment were obtained from the tumor resource library of SYSUCC. The serum copper level of the participants was measured using the Copper Assay Kit (PAESA chromogenic method) of the Roche cobas 8,000 system (BSBE, Beijing, China).

### Follow-Up and Endpoints

Patients were monitored every 3 months during the first 2 years, every 6 months during the subsequent 5 years, and once a year thereafter. The monitoring assessments included routine hematological and laboratory tests, menopausal status, breast and abdominal ultrasonography or computed tomography. X-ray and bone scans were performed annually.

The primary endpoint was disease-free survival (DFS), which was defined as the time from the date of diagnosis to the date of first sign of disease progression or death due to any cause. The secondary endpoint was overall survival (OS), which was defined as the time from the date of diagnosis to the date of death due to any cause.

### Statistical Analysis

Age was listed as mean with range, and categorical variables were represented as frequencies with percentages. The optimal cut-off value of serum copper level was determined by the maximally selected log-rank statistics using the “*maxstat”* package of R software (Hothorn and Zeileis, 2008). X-tile analysis of the 10-years OS was also conducted using the survival data of patients in the training cohort to decide the best cut-off point for serum copper level (Camp et al., 2004). Survival curves were estimated using the Kaplan-Meier method and compared with the log-rank test between the copper-high group and copper-low group. Variables achieving a *p* value <0.05 in the univariate Cox regression analysis could enter the backward stepwise multivariate Cox proportional hazards model, which has been tested on basis of the Schoenfeld residuals (Wileyto et al., 2013), and their hazard ratios (HRs) with 95% confidence intervals (CIs) were estimated. A predictive model incorporating serum copper and other independent clinicopathological factors identified from the multivariate Cox regression analysis was established and graphically presented as a nomogram, its discriminative performance and predictive accuracy were evaluated using the concordance index (C-index), which ranges from 0.5 (random chance) to 1.0 (perfect prediction), and calibration curves, whose Y-axis represents the actual observation of the survival rate, X-axis represents the survival rate predicted by the established nomogram, in both the training cohort and theSYSUCC-001 cohort. A two-sided *p value <* 0.05 was considered statistically significant. All statistical analyses were performed using R software (“rms” package, version 4.0.1; Vanderbilt University, Nashville, TN).

## Results

### Clinicopathological Characteristics of Patients in the Training Cohort

**FIGURE 1:**
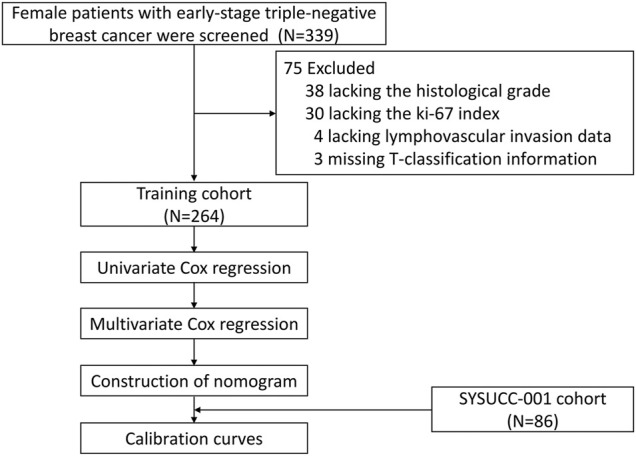
Flow chart of patient selection in this study.

After removing 75 patients with incomplete information, including 30 patients without Ki-67 index data, 38 patients without histological tumor grades, 4 patients without lymphovascular invasion data, and 3 patients without T-stage information (Figure 1), a total of 350 women with early-stage TNBC were eligible in the final analysis, including 264 patients in the training cohort, 86 patients in the SYSUCC-001 cohort. Specific patient clinicopathological characteristics in the training and SYSUCC-001 validation cohorts are shown in Table 1. In the training cohort, the mean age was 48 years [interquartile range (IQR), 41–57 years]. 169 (64.0%) women were premenopausal. Majority patients (98.9%) were pathologically diagnosed with invasive ductal carcinoma, and over half patients (56.1%) had tumors with a histological grade of 3. T1, T2, T3, and T4 tumors were presented in 90 (34.1%), 145 (54.9%), 23 (8.7%), and 6 (2.3%) patients, respectively. More than half the women (58.0%) were staged at N0, while the N1, N2, and N3 stages were present in 60 (22.7%), 27 (10.2%), and 24 (9.1%) women, respectively. In the SYSUCC-001 cohort, the mean age was 43.5 years (IQR, 37–51 years). Premenopausal women accounted for 79.1% (68). All patients were pathologically diagnosed with invasive ductal carcinoma, and 69 (80.2%) patients were histologically graded at 3. T1, T2, T3, and T4 tumors were showed in 29 (33.7%), 53 (61.6%), 2 (2.3%), and 2 (2.3%) patients, respectively. 52 (60.5%) women were staged at N0, and the N1, N2, and N3 stages were present in 19 (22.1%), 6 (7.0%), and 9 (10.5%) women, respectively.

**TABLE 1:** Characteristics of patients eligible in this study.

Characteristic	Number of cases (%)	
	Training cohort (*N* = 264)	SYSUCC-001 cohort (*N* = 86)
Age (years) at diagnosis	—	—
≤50	151 (57.2)	64 (74.4)
>50	113 (42.8)	22 (25.6)
Median	48	43.5
Interquartile range	41–57	37–51
Menopausal status	—	—
Premenopausal	169 (64.0)	68 (79.1)
Postmenopausal	95 (36.0)	18 (20.9)
Histological type	—	—
Invasive ductal carcinoma	261 (98.9)	86 (100.0)
Others	3 (1.1)	0
Histological grade^a^	—	—
1	6 (2.3)	1 (1.2)
2	110 (41.7)	16 (18.6)
3	148 (56.1)	69 (80.2)
Lymphovascular invasion	—	—
No	209 (79.2)	71 (82.6)
Yes	55 (20.8)	15 (17.4)
Ki-67 index at diagnosis <30%^b^	—	—
No	201 (76.1)	74 (86.0)
Yes	63 (23.9)	12 (14.0)
T stage^c^	—	—
1	90 (34.1)	29 (33.7)
2	145 (54.9)	53 (61.6)
3	23 (8.7)	2 (2.3)
4	6 (2.3)	2 (2.3)
N stage^c^	—	—
0	153 (58.0)	52 (60.5)
1	60 (22.7)	19 (22.1)
2	27 (10.2)	6 (7.0)
3	24 (9.1)	9 (10.5)
Serum copper (μmol/L)^d^	—	—
≤21.3	237 (89.8)	84 (97.7)
>21.3	27 (10.2)	2 (2.3)

a Histological grade at diagnosis was based on the degree of histological tumor differentiation.

b The Ki-67 index at diagnosis indicates DNA, synthetic activity as measured using immunocytochemistry.

c Diagnosed based on the AJCC, 2016 criteria (eighth edition).

d Cut-off values were determined using maximally selected log-rank statistics.

### Optimal Cut-Off Value of Serum Copper Level in the Training Cohort

**FIGURE 2:**
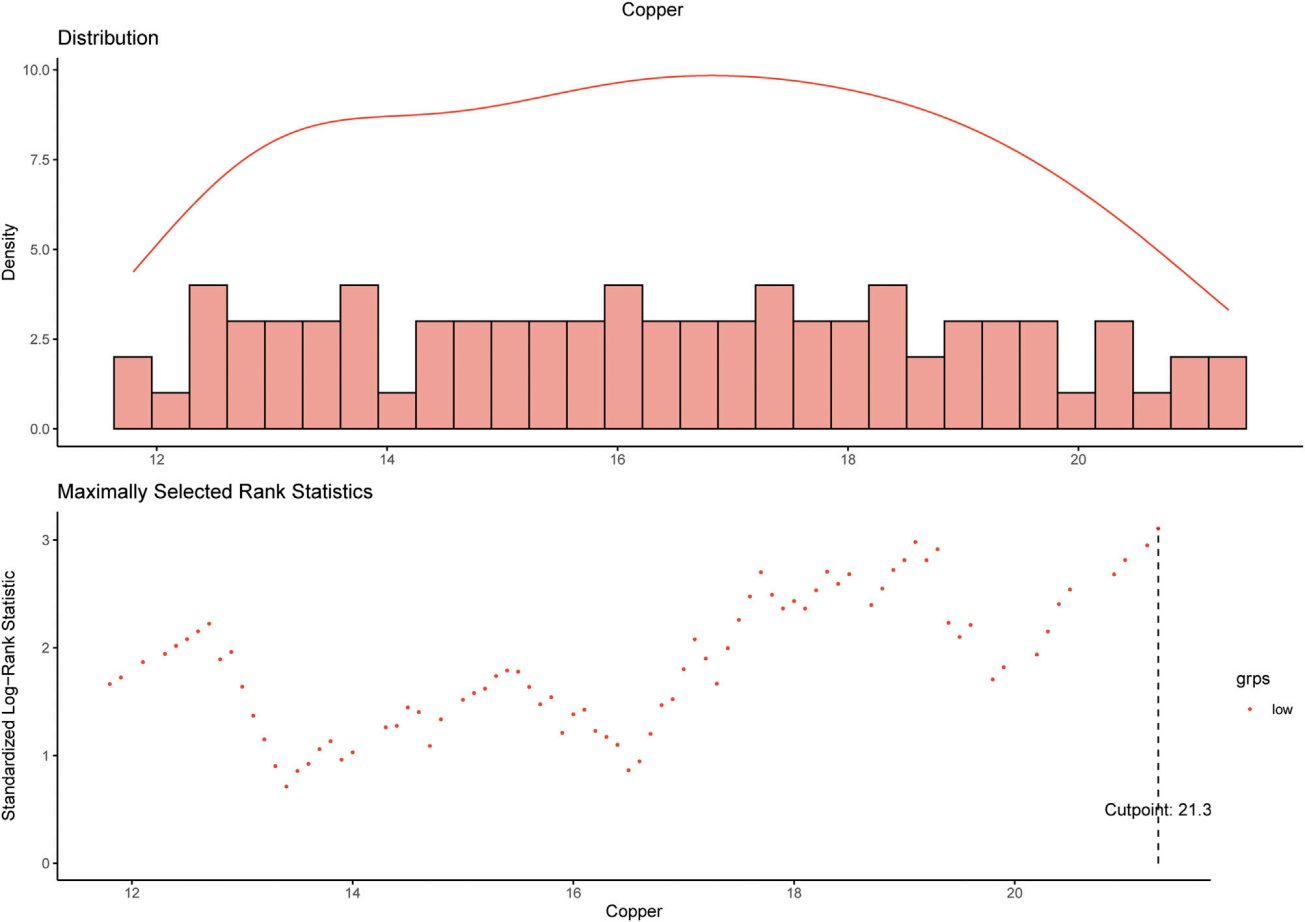
Determination of the cut-off value of 21.3 μmol/L for serum copper level by using maximally selected log-rank statistics.

Using maximally selected log-rank statistics, we determined that a cut-off point of 21.3 μmol/L for the baseline serum copper level would maximally divide patients into two copper-stratified groups (Figure 2), i.e., the copper-high group and the copper-low group. Consistently, the X-tile analysis based on the 10-years OS (Supplementary Figure S1) also resulted in a same cut-off serum copper level of 21.3 μmol/L (χ^2^ log-rank value, 11.5172). In the training cohort, the serum copper level was ≤21.3 μmol/L among 237 (89.8%) women and >21.3 μmol/L among 27 (10.2%) women. In the SYSUCC-001 cohort, 84 (97.7%) TNBC patients had a value ≤21.3 of the serum copper and 2 (2.3%) patients had the serum copper level >21.3 μmol/L.

### Survival Outcomes

After a median follow-up of 87.1 months (interquartile range, 67.6–113.4 months), we observed 64 DFS events and 51 deaths in training cohort, whose estimated 5-years DFS rates and 5-years OS rates were 79.6% (95% CI 74.8–84.6) and 83.7% (95% CI 79.3–88.4), respectively (Supplementary Figure S2).

**FIGURE 3:**
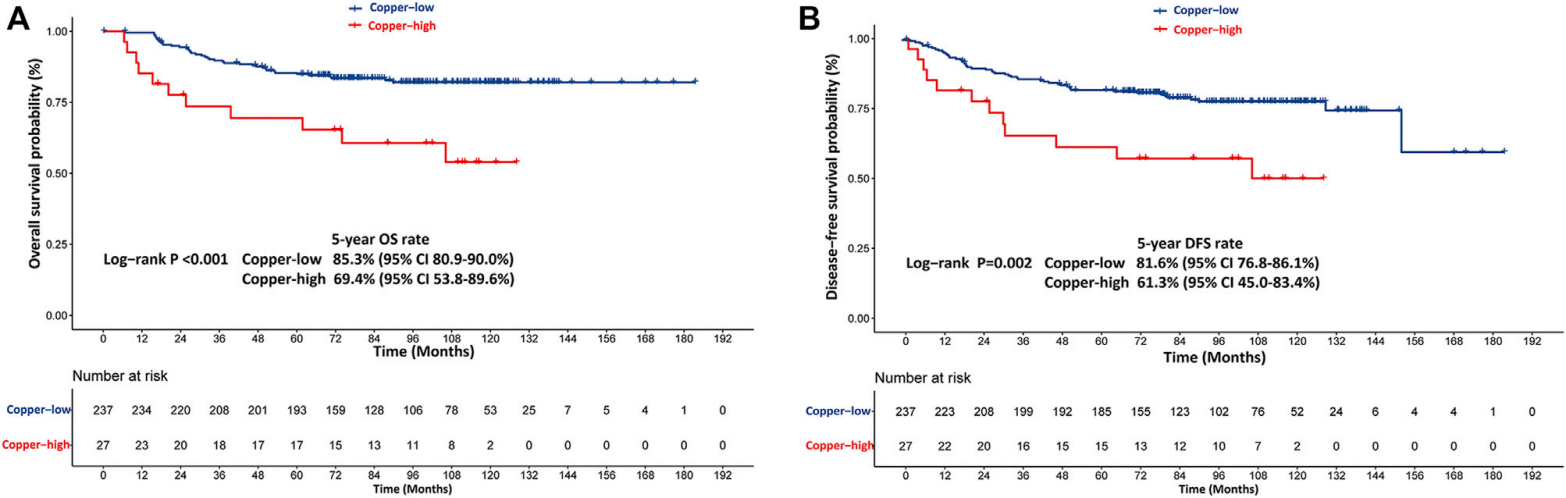
Kaplan-Meier survival curves stratified based on the baseline serum copper level in the training cohort. **(A)** Kaplan-Meier estimates of disease-free survival (DFS) probability. **(B)** Kaplan-Meier estimates of overall survival (OS) probability. Copper-low level: ≤21.3 μmol/L, Copper-high level: >21.3 μmol/L.

In the training cohort, patients with high copper levels had significantly worse DFS (*p* = 0.002) and OS (*p* < 0.001) than patients with low copper levels (Figure 3). The estimated 5-years DFS rates in the copper-high and copper-low groups were 61.3% (95% CI: 45.0–83.4) and 81.6% (95% CI: 76.8–86.1), respectively (Figure 3A). The estimated 5-years OS rates in the copper-high versus copper-low groups were 69.4% (95% CI: 53.8–89.6) versus 85.3% (95% CI: 80.9–90.0) (Figure 3B).

### Establishment of the Prognostic Model

**FIGURE 4:**
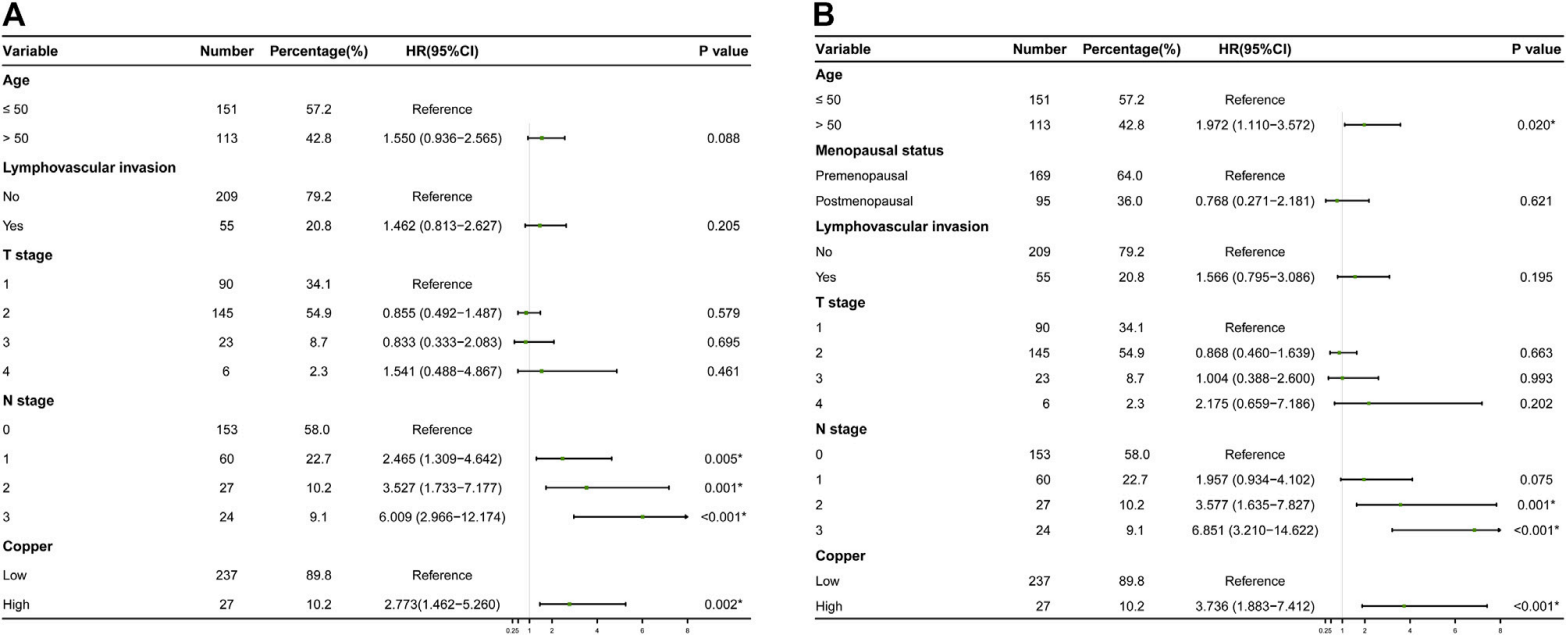
Results of final multivariate Cox regression analyses in the training cohort are represented as forest plots. **(A)** Forest plot of disease-free survival (DFS) probability. **(B)** Forest plot of overall survival (OS).

**FIGURE 5:**
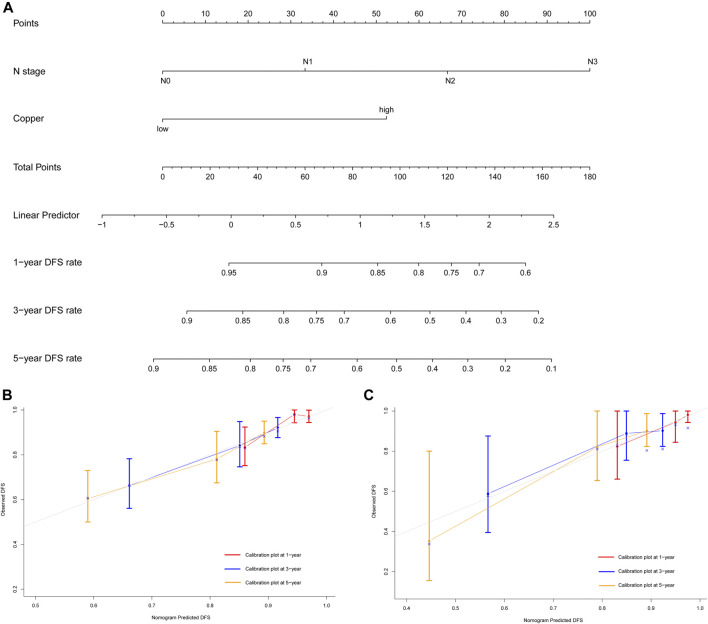
Development and validation of prognostic model for individualized prediction of disease-free survival (DFS). **(A)** Nomogram of current prognostic model for patients with early-stage triple-negative breast cancer. **(B)** Calibration curves for predicting DFS at 1-, 3-, and 5-years in the training cohort. **(C)** Calibration curves for predicting DFS at 1-, 3-, and 5-years in the SYSUCC-001 cohort.

The results of the univariate Cox regression analyses of DFS and OS in the training cohort are shown in Table 2, which identified following indicators associated with DFS among women with early-stage TNBC: age, lymphovascular invasion, T stage, N stage and serum copper level. Further multivariate Cox regression analysis demonstrated that N stage and serum copper level remained independent factors for DFS (Figure 4A). Based on the above two independent prognostic indicators, a prognostic model for individualized prediction of DFS was constructed and represented as a graphical diagram (Figure 5A).

**TABLE 2:** Univariate Cox regression analysis of disease-free survival and overall survival in women with breast cancer in the training cohort.

Characteristics	Disease-free survival			Overall survival	
	Hazard ratio (95%CI)		*p* value	Hazard ratio (95%CI)	*p* value
Age (year)					
≤50	Reference		—	Reference	—
>50	1.643 (1.004–2.687)		0.048*	2.063 (1.181–3.604)	0.011*
Menopausal status					
Premenopausal	Reference		—	Reference	—
Postmenopausal	1.563 (0.955–2.559)		0.076	1.815 (1.048–3.144)	0.033*
Histological grade^a^	—		—	—	—
1/2	Reference		—	Reference	—
3	1.140 (0.694–1.873)		0.605	1.305 (0.743–2.289)	0.354
Lymphovascular invasion					
No	Reference		—	Reference	—
Yes	2.495 (1.481–4.205)		0.001*	2.728 (1.542–4.827)	0.001*
Ki-67 index at diagnosis <30%^b^					
No	Reference		—	Reference	—
Yes	0.999 (0.565–1.761)		0.996	0.836 (0.429–1.630)	0.600
T stage^c^					
1	Reference		—	Reference	—
2	0.870 (0.505–1.499)		0.616	0.863 (0.464–1.607)	0.642
3	1.079 (0.437–2.663)		0.868	1.384 (0.546–3.511)	0.494
4	3.529 (1.213–10.265)		0.021*	4.905 (1.647–14.605)	0.004*
N stage^c^					
0	Reference		—	Reference	—
1	2.537 (1.348–4.774)		0.004*	1.976 (0.943–4.138)	0.071
2	3.633 (1.786–7.392)		<0.001*	3.665 (1.677–8.009)	0.001*
3	6.328 (3.150–12.711)		<0.001*	7.099 (3.375–14.932)	<0.001*
Serum Copper (umol/L)^d^					
≤21.3	Reference		—	Reference	—
>21.3	2.560 (1.363–4.811)		0.003 *	3.006 (1.542–5.861)	0.001*

^*^ *p*＜0.05.

a Histological grade at diagnosis was based on the degree of histological tumor differentiation.

b The Ki-67, index at diagnosis indicates DNA, synthetic activity as measured using immunocytochemistry.

c Diagnosed based on the AJCC 2016 criteria (eighth edition).

d Cut-off values were determined using maximally selected log-rank statistics.

**FIGURE 6:**
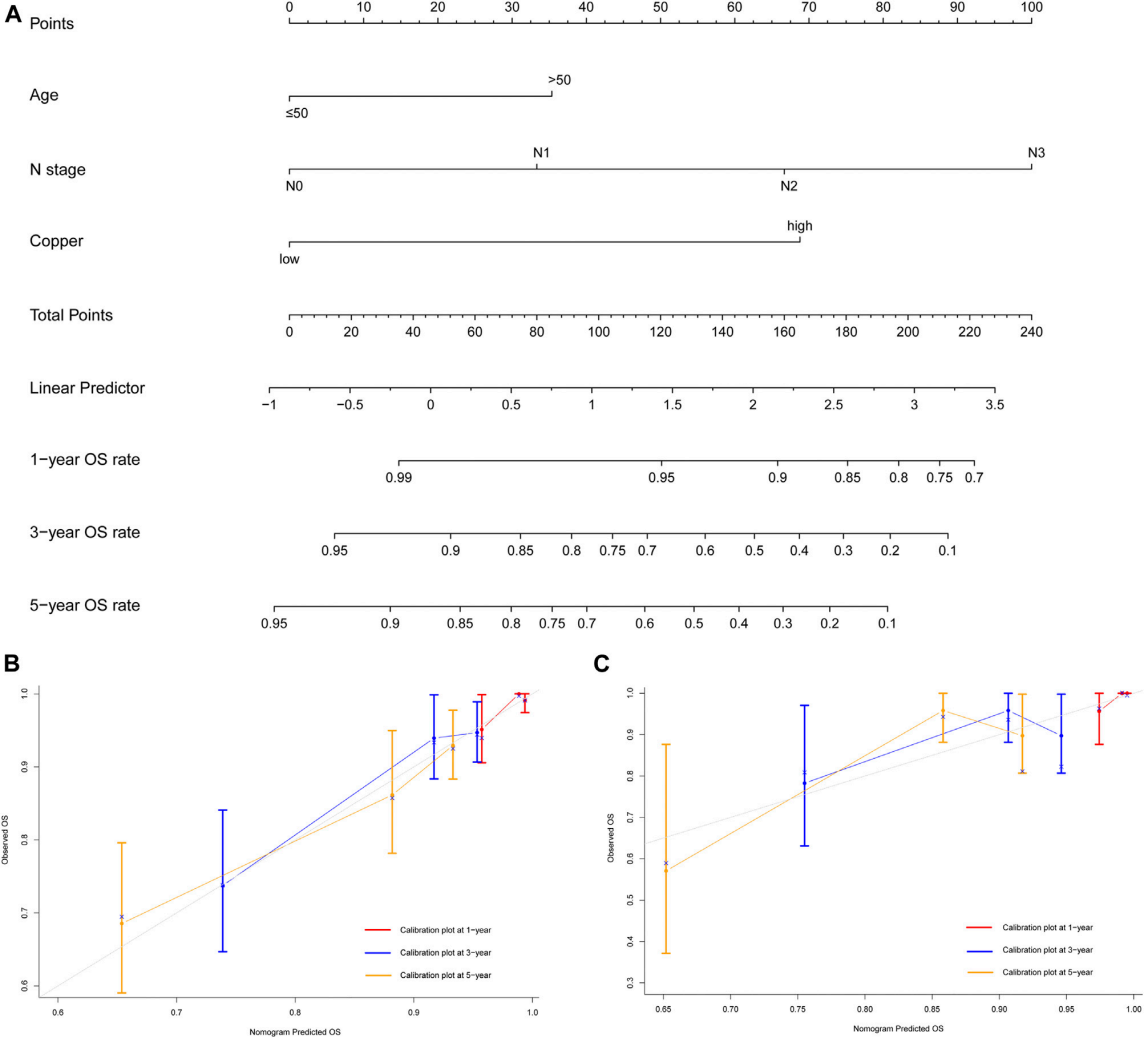
Development and validation of prognostic model for individualized prediction of overall survival (OS). **(A)** Nomogram of current prognostic model for patients with early-stage triple-negative breast cancer. **(B)** Calibration curves for predicting OS at 1-, 3-, and 5-years in the training cohort. **(C)** Calibration curves for predicting OS at 1-, 3-, and 5-years in the SYSUCC-001 cohort.

Variates achieving the predetermined significance level (*p* < 0.05) in the univariate Cox regression analysis of OS in the training cohort (Table 2), including age, menopausal status, lymphovascular invasion, T stage, N stage and serum copper level, were furtherly analyzed in the multivariate Cox regression analysis (Figure 4B), which revealed that age, N stage and serum copper level were still significantly associated with OS. Using these three independent prognostic indicators, we developed a prognostic model for the individualized prediction of OS (Figure 6A). Through our nomogram models, clinicians and patients could individually predict patients’ prognosis, for example, patients with high serum copper levels assign a high score both on its point scale and on the total point scale, which determinates a poor prognosis, additional care and frequently monitoring are essential for them.

### Evaluation of the Performance of the Prognostic Model

The prognostic nomogram of DFS showed excellent discriminative ability and predictive accuracy. The C-index after bootstrap correction was 0.689 (95% CI 0.621–0.757) in the training cohort and 0.704 (95% CI 0.577–0.831) in the SYSUCC-001 cohort. Calibration curves for the DFS probability at 1-, 3-, and 5-years in the training and SYSUCC-001 cohorts both demonstrated satisfactory consistency between the nomogram-predicted and actual survival (Figures 5B,C).

The prognostic nomogram of OS also showed satisfactory discrimination, with an excellent C-index of 0.728 (95% CI 0.654–0.802) in the training cohort and 0.653 (95% CI 0.487–0.819) in the SYSUCC-001 cohort. The calibration curves for the probability of OS at 1-, 3-, and 5-years (Figures 6B,C) also suggested good agreement between the observed and nomogram-predicted survival.

## Discussion

In current study, we found that patients with early-stage TNBC were a heterogeneous population with different serum copper levels. According to maximally selected log-rank statistics, we determined that 21.3 μmol/L was the best cut-off value of serum copper to stratify patients into two different groups. Moreover, multivariate Cox analysis identified high serum copper level as an independent factor influencing survival. Prognostic nomograms incorporating serum copper level and other clinicopathological factors were developed, which showed excellent discrimination and good consistency between the nomogram-predicted survival and actual observations in both the training and SYSUCC-001 cohorts.

As a catalytic cofactor and a structural component of various proteins and enzymes, copper is indispensable for organisms, and its homeostasis is crucial for many physiological functions (Denoyer et al., 2015; Setty et al., 2008; Bhattacharjee et al., 2017; Juloski et al., 2020; Shanbhag et al., 2019; Chan et al., 2020). Copper dysregulation and variations in copper metabolism-related proteins have been found in many tumors, including breast cancer (Terada et al., 1999; Gupte and Mumper, 2009; Hlynialuk et al., 2015; Juloski et al., 2020). The multifaceted effect of copper on carcinogenesis has been confirmed, it not only interrupts the initiation and progression of tumors but also promotes cell proliferation, angiogenesis, cell growth, and cell migration (Brady et al., 2014; Angelé-Martínez et al., 2017; Lelièvre et al., 2020). Thus, copper chelators or ionophores that deplete or increase the copper level in tumors have been explored in preclinical studies, and been reported to have promising clinical value (Chan et al., 2017; Xu et al., 2018; Chan et al., 2020; Cui et al., 2021).

In 1975, Schwartz firstly reported the potential value of copper in the diagnosis and prognostication of tumors (Schwartz 1975). Gupte and Mumper recently found increased serum copper levels and oxidative stress in multiple malignancies, these factors are the hallmarks of a range of cancers, and may thus provide a basis for the development of potential novel anti-cancer treatments, such as copper chelators (Gupte and Mumper, 2009). A study of patients with hematological malignancies demonstrated that serum copper levels declined and even normalized during tumor remission, but rebounded to the pre-treatment levels when relapses occurred (Kaiafa et al., 2012). High serum copper levels have also been confirmed to be associated with tumor progression, staging, and chemotherapy resistance among patients with metastatic colon, advanced breast, and lung cancers, serum copper levels among non-responders were approximately 130–160% of that in responders (Denoyer et al., 2015; Majumder et al., 2009; Lelièvre et al., 2020). Given the short half-life of copper (Lelièvre et al., 2020), and its central role in cancer development from tumorigenesis to metastasis, we think that serum copper level may be a promising biomarker for prognostication and the monitoring of treatment efficacy.

However, some studies on breast and colon cancers have reported that the serum copper level was lower in tumor tissues than in normal tissues (Lelièvre et al., 2020; Kucharzewski et al., 2003). Leila et al. also did not support the association between copper level and breast cancer in their meta-analysis, but the heterogeneity among the original studies enrolled in this meta-analysis should be noted (Jouybari et al., 2020), which is similar to other meta-analyses on Hodgkin lymphoma and lung cancer (Mahabir et al., 2010; Thompson et al., 2010). At present, there is no consensus on the relationship between serum copper level and cancers, and studies investigating this associations especially in early-stage TNBC remain rare. Thus, it is necessary to explore the relationship between serum copper and TNBC.

Based on the stratification of serum copper levels, we established prognostic models that incorporated the serum copper level and other clinicopathological factors, including age and N stage, which is an indicator of metastasis (Amin et al., 2017). Compared with the previous 21-gene, MammaPrint, and PAM50 predictive models, our nomograms are more economical and easily applicable in clinical practice. The 21-gene test is limited to lymph node-negative patients with early-stage ER+/PR+, HER2-breast cancer, its prognostic performance in other breast cancer subtypes and in lymph node-positive breast cancers is poorly understood (Poorvu et al., 2020). PAM50 was developed based on the mRNA levels of 50 genes in order to discriminate intrinsic breast cancer subtypes and stratify relapse risks, the C-index of its prognostic value is 0.63–0.67 (Gnant et al., 2015; Wallden et al., 2020). MammaPrint is a diagnostic assay based on 70 genes and evaluates the distant recurrence risk in early-stage breast cancer. This test has been validated in a real-world study, wherein it achieved a C-index of 0.614 (Ibraheem et al., 2020). These findings suggest that our prognostic models have a better predictive accuracy. Additionally, despite the TNM staging system, several predictive models were explored according to inflammatory status, tumor marker, stromal tumor-infiltrating lymphocytes, gene signature and so on with C-index from 0.69 to 0.77 (Shi et al., 2019; Yang et al., 2019; Zheng et al., 2020), compared with these models, our predictive nomogram achieved a comparative prognostic accuracy, and is more economic and convenient. Moreover, our study innovatively proposes a prognostic model in view of the impact of trace element copper with clinicopathological factors.

There are some limitations to our study. Firstly, as a retrospective study, selection bias and incomplete data were inevitable, an external validation rather than a same-institute validation might enhance the power of our predictive models. We minimized the risk of selection bias by enrolling all eligible patients to date and validating our results in a relatively separate cohort. Secondly, the serum cooper might be influenced by food, but we failed to obtain and explore information of patients’ dietary, so a prospective study to control these potential confounding factors and validate the prognostic value of our nomograms is warranted in future. Thirdly, we only explored the relationship between the baseline serum copper level at the time of diagnosis and early-stage TNBC. We did not measure the serum copper levels in healthy control subjects without TNBC, and monitor the dynamic changes in serum copper levels during the treatment of TNBC. Fourthly, this study was limited to patients from China, so the extension of our results to patients from other geographic regions or with other racial backgrounds should be done with caution. Finally, fundamental cytological studies and investigations of the underlying mechanisms are necessary to further explore the usefulness of serum copper levels in early-stage TNBC.

In conclusion, we proposed an optimal cut-off value of baseline serum copper levels to maximally distinguish women with early-stage TNBC into different risk groups in current study. Based on this serum copper level cut-off, we developed and validated prognostic models for personalized survival predictions, resulting in satisfactory discrimination and good agreement between the nomogram-predicted and actual survival for survival in training and validations cohorts.

## Data Availability

The datasets presented in this study can be found in online repositories. The names of the repository/repositories and accession number(s) can be found below: Research Data Deposit public platform (www.researchdata.org.cn), with the approval RDD number, RDDA2021002097.

## Supplementary Material

Supplementary Figure S1 Determination of the cut-off value of serum copper level and survival analyses. X-tile analysis of 10-year OS was performed using the patients’ data to identify the best cut off value for serum copper level. The optimal cut-off points highlighted by the black circles in the left panels are shown as histograms of the entire cohort (middle panels), and Kaplan-Meier plots are displayed in the right panels. The optimal cut-off value for serum copper level was 21.3 μmol/L (χ2 = 11.5172, P = 0.0189).

Supplementary Figure S2 Kaplan-Meier survival curves with log-rank test in the training cohort. A: Kaplan-Meier estimates of disease-free survival (DFS) probability. B: Kaplan-Meier estimates of overall survival (OS) probability.
