# Supplementary figures and images for: Establishment and Validation of Prognostic Nomograms Based on Serum Copper Level for Patients With Early-Stage Triple-Negative Breast Cancer
Source: Front Cell Dev Biol. 2021 Nov 25;9:770115. doi: 10.3389/fcell.2021.770115 (PMC8657150; doi:10.3389/fcell.2021.770115)

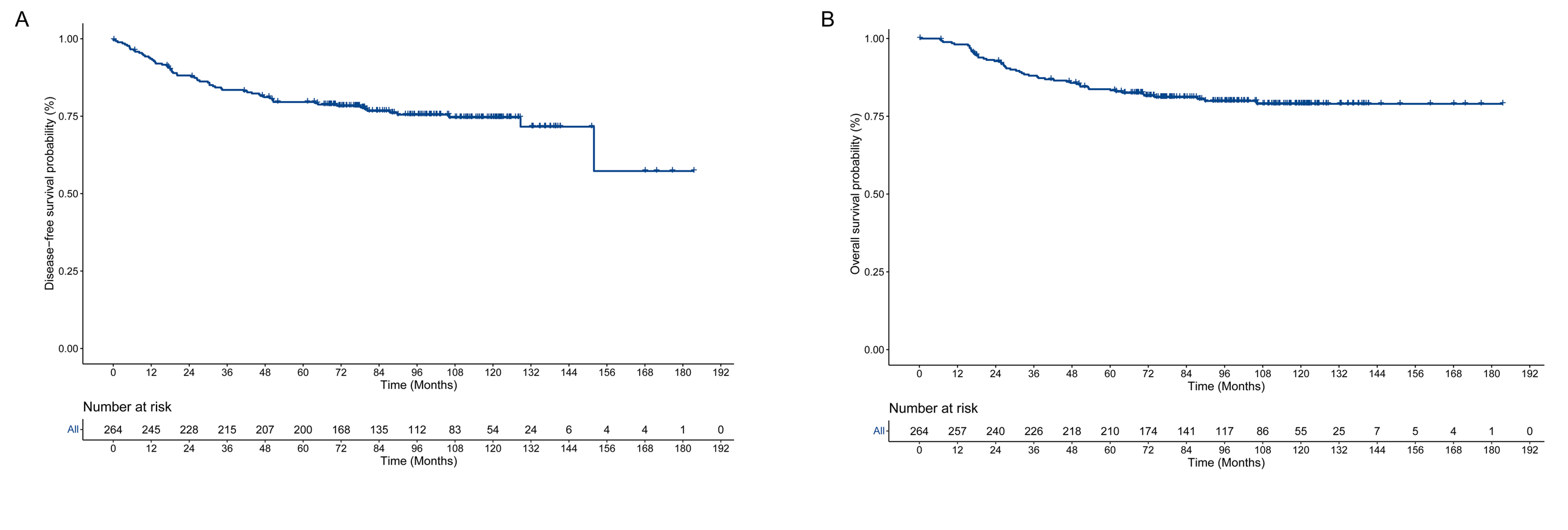

Supplement: Supplementary file 1 [file Image2.TIF]

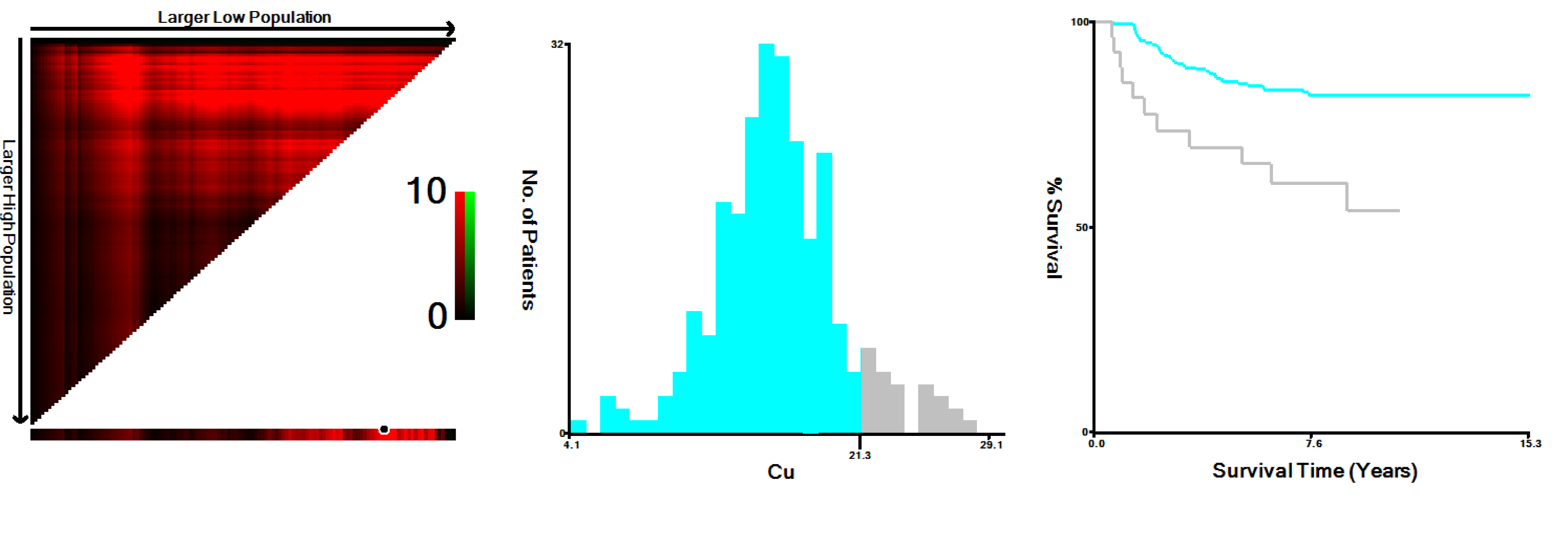

Supplement: Supplementary file 2 [file Image1.TIF]
